# Core, Evidence Application, Research (CEAR): a Theory to Frame Separate Skills for Evidence-Based Practice and Research in Medical Education

**DOI:** 10.1007/s40670-026-02730-7

**Published:** 2026-04-21

**Authors:** Rosanna P. Watowicz, Rosa K. Hand

**Affiliations:** 1 Department of Nutrition, School of Medicine, Case Western Reserve University, Cleveland, OH USA; 2 Office of Medical Student Research and Scholarship, School of Medicine, Case Western Reserve University, Cleveland, OH USA

**Keywords:** Evidence-based practice, Medical education, Research education, Physician scientist

## Abstract

Research is embedded into the curriculum of most US medical schools. However, there is a great deal of variety in the curricular approaches to teaching research. Further, some programs choose to focus on teaching the conduct of research while others focus on the application of research findings (i.e. evidence-based practice). This variation may lead to conflation of two independent skill sets. This investigation advances a theory (called CEAR—Core, Evidence Application, Research) that research skills and evidence-application skills are independent from one another. We evaluated the CEAR model using a pre-post design and paired t-tests at a medical school with defined curricula in both research and evidence-based practice. We found that medical student skills were low in CEAR model Core skills (approximately 42% of possible maximum score on the CEAR tool at baseline). Core and Research skills increased at each timepoint, although whether the changes were significant varied by research subdomain. Evidence-application scores improved after evidence-based practice activities within the curriculum (22.8 ± 16.4% to 30.2 ± 18.4% *p* < 0.001), but did not increase significantly further after the research experience (30.2 ± 18.4% to 36.5 ± 17.3%, *p* = 0.126). Limitations include the single medical school design and the limited number of students who completed all three data collection timepoints. Overall, we believe CEAR is a valid model for medical educators to use to conceptualize the separation of research and evidence application, the core skills required for both, and the sub-domains, in their development of curricula. Additionally, the CEAR tool may be useful for student assessment and curricular evaluation.

## Introduction

Research is embedded into the curriculum/experience of most US medical schools with the dual goals of increasing the number of physician scientists and teaching students to apply research in practice (i.e., evidence-based practice) [1]. The Liaison Committee on Medical Education (LCME) recognizes the importance of these skills and goals, including two elements directly related to the teaching of research (3.2 Community of Scholars/Research Opportunities and 7.3 Scientific Method/Clinical/Translational Research) [2]. Other LCME elements are connected to research more peripherally, including 4.3 scholarly productivity, 6.3 self-directed and life-long learning, 7.4 critical judgement/problem solving skills, 7.7 medical ethics and 7.8 communication skills [2]. Research conduct offers an opportunity for students to develop the foundational skills outlined by LCME, explore specialties, build relationships with faculty mentors, and differentiate themselves for residency.

The importance of medical student research in residency matching has increased over the past two decades. According to data from the 2022 National Residency Match Program, matched plastic surgery candidates had an average of 28.4 presentations, abstracts, and/or publications, followed closely by neurological surgery (25.5) and dermatology (20.9) [3]. These are exponential increases from 2009, when matched candidates in the same specialties had an average number of listed outputs of 8.2, 7.8 and 7.2, respectively [3]. Even specialties not traditionally considered competitive have increasing expectations for research outputs to match (e.g. family medicine 4.1, pediatrics 5.6, emergency medicine 5.1) [3]. This trend is expected to continue since the evolution of step exams from a scored to a pass/fail format.

However, despite these high quantitative research outputs, the quality of the experiences and outputs are unclear. 59% of medical student authored articles are never cited, suggesting that the scholarly community finds them of little value [3]. Medical schools differ on whether they teach the LCME elements by asking students to do research, to propose research, or to critique research [4]. Schools also vary on the design of their research curriculum, ranging from mandatory to optional, timing across the curriculum, and final assessments of oral and/or written work [1, 5]. There is no standardized way that medical schools measure research or evidence application skills or knowledge, or that they evaluate the goals or effectiveness of these curricular goals. Further, there are very few tools that measure all four As of the evidence-based practice process (i.e. Ask, Acquire, Appraise, and Apply), and none to our knowledge that assess both research and evidence application together [6].

We have argued previously that the two stated goals of teaching research (increasing physician scientists and confident application of research) are, in fact, very different skills [7]. Medical schools that require students to conduct research are aligning more with the first goal, and those that require critiquing are aligned with the second goal. Elessi et al. [8] have made a similar argument and describe an approach to teaching research critique with the explicit goal of understanding not how to conduct research but how to apply the findings of research into practice.

We previously developed a model that illustrates this theory—that research conduct and evidence application are two different sets of skills that build upon a shared set of core skills required for both. We call this the Core, Evidence Application, and Research (CEAR) model [7]. The CEAR model has been validated in a group of registered dietitians but we believe it can be useful in other health professions including medicine, and in learners as well as practitioners. The CEAR model fills a gap in the current understanding of research versus evidence-based practice. Beyond CEAR, there is no existing validated framework to differentiate research skills from evidence-based practice skills in medical students or practitioners, nor is there a framework for how they related to one another.

The purpose of this study was to further examine the validity of the CEAR model as a theory to discuss, plan, and assess research and evidence-application education for medical students using a pre-test, post-test design. If CEAR model scores increase as we assume they would in response to research and evidence-application curricula, this demonstrates the validity of the CEAR model in medical students. Further, we would expect scores in the Core, Research, and Evidence Application domains to group together for individual students, validating the theory that the domains are unique from one another.

## Materials and Methods

For this validation study, we measured self-reported experience in research and evidence-application skills for medical students before and after participating in research and evidence-based practice curricula. We hypothesized that, if the CEAR tool is valid, CEAR total and domain scores would improve following these curricula that include explicit evidence-based practice and research conduct learning objectives.

### CEAR Model, Survey Tool, and Scoring

The original development of the CEAR model is described elsewhere [7], but briefly, we compiled research and evidence-based practice competencies from existing research and professional documents. Because our model was initially focused on registered dietitians, some of the documents were from dietetics-specific accrediting bodies, however none of the skills included in the final, previously validated CEAR model and tool are profession-specific. After curating these documents, we then honed the list by combining competencies that were similar to each other and, finally, three authors independently categorized the competencies as Core, Research, or Evidence Analysis. After independent categorization, we discussed the results and came to agreement.

The CEAR model is a framework for understanding the relationship between research and evidence-based practice. It represents research conduct and evidence application as two separate sets of skills with shared “Core” skills as the foundation of both. The CEAR tool operationalizes the CEAR model. The CEAR tool is made up of 68 total items, each item representing a discrete skill within a particular CEAR domain or sub-domain. The choices for each item are on a five-point scale (0 = ‘I have never learned this skill’; 1 = ‘I have learned this skill but not performed it’; 2 = ‘I have performed this skill under supervision/mentorship’; 3 = ‘I have performed this skill independently’; 4 = ‘I have mentored others in this skill’) and participants self-report their experience level. The CEAR tool can be scored as a total CEAR score (68 items, total maximum score of 272), or as domain scores (Core, Research, and Evidence Application). Domain scores are a sum of ratings for all skills assigned to the domain. The Core domain has a total of 8 items with a maximum domain score of 32. The Evidence Application domain has a total of 9 items and a maximum total domain score of 36. The research domain has a total of 51 items with a maximum total domain score of 204. The Research domain further contains sub-domains: planning, administering, conducting, disseminating, and secondary research, which can each be scored individually as well. Given the variable number of items per domain/subdomain, scores can also be calculated as a percentage of possible maximum.

We treated the CEAR tool as continuous, based on the guidance that, when normality is confirmed, Likert-type response options can be treated as continuous data [9] and that when several Likert-type response options are grouped together in a scale, means are an appropriate measure of central tendency [10] The CEAR tool used for this study was identical to the CEAR tool used in the validation study for registered dietitians, as we believe the included skills are relevant to all healthcare professionals.

### Background Curriculum

At the Case Western Reserve University School of Medicine, an allopathic medical school at a research-intensive, private, Midwest university, we require students to conduct research, culminating in a written thesis, and also have an explicit curriculum teaching students to critique and apply research, i.e. evidence application [11–13].

During their first year of medical school, students complete 28 h of a didactic research curriculum (plus additional corresponding out of class work) intended to expose students with a variety of backgrounds and interests to important biomedical research methodologies and norms including responsible conduct of research, protection of human subjects, statistical analysis, and scientific dissemination. The didactic curriculum concludes in May of the first year, at which point students begin a full time, immersive, 12-week mentored research experience. In this experiential learning component, students work with a faculty researcher and, under mentorship, carry out, and report on a small research project. Students are given wide latitude as to the topic and type of research they conduct; from basic science (in silico, in vitro) to prospective or retrospective human studies, through health services research and big data or secondary research including systematic reviews and meta-analysis. Alternatively, students can choose to complete a scholarship project including quality improvement or medical education evaluation.

Additionally, during their first year of medical school, students participate in evidence-based practice activities, including evidence-application, within the basic science curriculum. This curriculum incrementally introduces students to the “four As” of evidence-based practice—ask, acquire, appraise, apply. Students incorporate these skills into their problem-based learning groups, which make up the majority of the curriculum. They initially learn to appraise literature based on assigned articles and then are taught to ask questions and eventually acquire their own literature, all while applying the findings to the problem based learning [14].

We used these curricula as an opportunity to further validate the CEAR model by comparing student CEAR scores [7] early in their first year, at the end of the first year (following a didactic research curriculum and explicit EBM curriculum) and in the middle of their second year (following both didactic curriculum elements, hands one research experience, and a culminating thesis).

### Data Collection

We recruited medical students who had completed the CEAR tool, a measure of the CEAR model, as a self-assessment during the first semester of their first year of medical school, before beginning their research curriculum. Baseline CEAR surveys were collected via Qualtrics online survey in the regular course of education in the Fall of 2023 and Fall of 2024 (the first semester of medical school for students in the class of 2027 and 2028, respectively). Students were provided with the survey link and asked to complete the CEAR tool during the second research class period. These surveys were initially intended to be used solely as a self-assessment of research and evidence-application skills for the medical students to consider what skills they might like to expand in their full-time research experience.

We subsequently received IRB approval (Case Western Reserve University IRB 20241458) to use these CEAR scores as baseline data for our study, and to prospectively follow-up with students for research purposes. Follow-up data were collected using the same online Qualtrics survey. For follow-up data collection, students were emailed the survey link. After reviewing the informed consent, students chose whether or not to complete the survey. Follow-up surveys were completed outside of class and students who completed the survey were compensated with a $10 gift card. In order to provide students with their individual results at baseline, and to match baseline data to follow up data, participant identifiers were collected on both. Data were deidentified once baseline and follow-up data were linked.

Each survey had between one and three attention checks—questions where the answer is pre-specified and participants simply need to select the answer which they are instructed to choose. We eliminated survey responses in which participants missed any of the attention check questions as these suggest they were not providing valid data but rather rushing through the survey providing random answers.

### Statistical Analysis

We compared baseline CEAR overall scores between students who had multiple valid timepoints to those who had only one timepoint (baseline) using two-sided t-tests to determine whether only students with a particular interest in research took the follow-up survey and to assess the risk of non-response bias. We also compared the two different class years at baseline using a t-test. In both instances we tested first for equal variances using Levene’s test.

Using baseline data from both classes combined, we determined the chronbach’s alpha for the overall tool and the domain scores.

We calculated means and standard deviations for the raw scores and the scores as a percentage of maximum possible, in all students with data at each timepoint. We then used paired t-tests to compare time 1 and time 2 data, time 2 and time 3 data, and time 1 and time 3 data. The number of students with paired data for each combination of timepoints varied from the overall students who had data at each singular timepoint, but the patterns in change in score were the same.

Statistical tests were conducted in SPSS version 29 (IBM Inc Armonk, NY) with *p* < 0.05 considered statistically significant.

## Results

The number of responses varied by timepoint, as shown in Table 1. Individuals who completed multiple timepoints did not have significantly different total CEAR scores to those who completed only one timepoint (*p* = 0.282). The two class years also had similar total CEAR scores at baseline (*p* = 0.102). Therefore, we proceeded with collapsing the data from both class years and analyzing it together.

The chronbach’s alpha for the total CEAR and the individual domains were all greater than 0.8, suggesting internal reliability and content validity [15] (Table 1).

**Table 1:** Chronbach’s alpha for CEAR overall and domain scores for both cohorts (*n* = 323) at time 1

Domain	Chronbach’s alpha
CEAR overall	0.950
Core Domain	0.806
Evidence Application Domain	0.869
Research Domain	0.938

CEAR total scores increased after didactic education (time 1 vs. time 2, 94.4 ± 34.7 to 109.6 ± 31.0, *p* < 0.001) and further after the research experience (time 3 = 132.9 ± 25.4; p 0.001). Similar trends were observed in the domain and sub-domain scores: scores improved significantly across all domains/subdomains between time 1 and 3 but after only the didactic education at time 2, the subdomain scores of conducting primary research, administering research, and research dissemination did not have significant changes. The Evidence Application Domain gains were statistically between time 1 and 2, as were the gains in the secondary research subdomain. At baseline, the highest score was in the Research Dissemination subdomain at approximately 49% of possible (Table 2). This is in comparison with the Core Domain (approximately 42% of possible at baseline) (Table 2), which we would anticipate should be highest. The improvements in the Core domain reflect some content taught in the research curriculum and some taught in the evidence-based practice activities. However, at time 3 Research Dissemination (62.8 ± 10.9%) was still slightly higher than Core (60.1 ± 12.8%).

**Table 2:** Changes in CEAR total and domain scores medical students after research and evidence-based practice curricular elements. Scores are presented as mean ± SD for both raw score, and parenthetically, percent of possible score. P-values reflect paired t-tests

	T1 *n* = 313	Didactic Research Curriculum; Explicit, hands-on EBM curriculum	T2 *n* = 51	12-week full time Research Experience	T3 *n* = 83	*P* value time 1 vs. time 2 *N* = 46	*P* value time 2 vs. time 3 *N* = 17	*P* value time 1 vs. time 3 *N* = 72
CEAR Total	94.4 ± 34.7 (34.7 ± 12.7%)		109.6 ± 31.0 (40.3 ± 11.4%)		132.9 ± 25.4 (48.9 ± 9.4%)	**< 0.001**	**0.001**	**< 0.001**
Core Domain	13.3 ± 4.8 (41.7 ± 15.0%)		17.3 ± 3.9 (54.1 ± 12.3%)		19.3 ± 4.1 (60.1 ± 12.8%)	**< 0.001**	**0.001**	**< 0.001**
Evidence Application Domain	8.2 ± 5.9 (22.8 ± 16.4%)		10.9 ± 6.6 (30.2 ± 18.4%)		13.2 ± 6.2 (36.5 ± 17.3%)	**0.005**	0.126	**< 0.001**
Research Domain	72.8 ± 27.9 (35.7 ± 16.7%)		81.4 ± 25.9 (40.0 ± 12.7%)		100.5 ± 20.0 (49.3 ± 9.8%)	**0.025**	**0.003**	**< 0.001**
Designing Primary Research SubDomain	18.9 ± 8.5 (39.3 ± 17.7%)		21.4 ± 7.8 (44.6 ± 16.2%)		26.4 ± 6.9 (54.9 ± 14.3%)	**0.024**	0.057	**< 0.001**
Conducting Primary Research SubDomain	15.8 ± 6.5 (39.5 ± 16.2%)		17.1 ± 5.6 (42.8 ± 13.9%)		21.0 ± 5.2 (52.4 ± 13.0%)	0.081	**0.034**	**< 0.001**
Administering Research SubDomain	5.0 ± 5.2 (15.6 ± 16.3%)		5.7 ± 5.0 (17.9 ± 15.5%)		6.9 ± 5.6 (21.6 ± 17.5%)	0.197	0.128	**< 0.001**
Research Dissemination SubDomain	27.4 ± 11.4 (48.9 ± 20.3%)		29.9 ± 11.7 (53.4 ± 20.9%)		35.2 ± 6.1 (62.8 ± 10.9%)	0.462	**0.015**	**< 0.001**
Designing and Conducting Secondary Research SubDomain	5.8 ± 4.9 (20.7 ± 17.6%)		7.2 ± 5.5 (25.8 ± 19.6%)		11.1 ± 6.5 (39.6 ± 23.3%)	**0.012**	0.148	**< 0.001**

## Discussion

Based on these analysis, the CEAR tool is valid for measuring core, research, and evidence application skills in medical students. The validity of the CEAR tool supports our CEAR model by demonstrating that research and evidence-application skills develop in response to different experiences/curricula. CEAR total, and domain scores in Core, Evidence Application and Research increased significantly in response to curricula and experiences in research and evidence-based practice. Incremental increases between timepoints were logical based on the curricula presented. For example, the incremental increase in Evidence Application was significant from time 1 to 2 but not time 2 to 3, which is logical because the explicit evidence-based practice curriculum takes place between time 1 and 2. Similarly the designing primary research subdomain increased significantly from time 1 to 2 but not time 2 to 3, aligning with the planning phase of research and the methodological focus of the didactic curriculum. Conducting research and dissemination subdomains increased significantly from time 2 to 3 only, which aligns with the experiential phase of the curriculum and the write-up of a completed project. The secondary research subdomain only increased from time 1 to 2, in response to a didactic session on systematic review and meta-analysis; however since only some students use these methods for their projects it is logical that few changes were seen from the experiential portion.

These findings add to our previous validation in a group of registered dietitians in two ways. First, our initial validation testing was performed at a single time point [7]. Data from the current study demonstrate that CEAR can detect change over time in response to known curricula. Second, validity among medical students provides evidence that CEAR may be useful across different healthcare disciplines.

Core domain scores started (approximately 42% of possible) and ended (approximately 60% of possible) surprisingly low considering that the CEAR model asserts that core skills are foundational in higher level research and evidence application skills. Similar patterns were seen in our initial validation with registered dietitians [7]. This suggests that medical educators may be incorrectly assuming that Core domain skills are taught in undergraduate education, thus skipping these foundational skills in medical school, and instead advancing directly to more advanced research and evidence application training. For example, previous research demonstrates that medical students are unwilling to accept uncertainty [16]; they may be more accepting of uncertainty if their Core domain skill of “evaluate available research evidence including consideration of validity, magnitude, and relevance and distinguish research evidence from opinion” had been reinforced in medical school.

Dissemination subdomain scores were the highest of all domain and subdomain scores (as a percent of maximum possible score) at both baseline and time 3. This suggests that when undergraduates or gap year students are participating in research prior to medical school, they may have substantial exposure to the publication process even if they have limited exposure to the project development and conceptualization. Additional possible interpretations include that CEAR does not adequately capture the basic science research skills that are developed by many undergraduate research experiences, leading to lower scores in other Research subdomains, or may reflect a high (if false) sense of competence [17] among medical students around their dissemination knowledge.

The incremental improvements seen after each component of the curriculum support the value of an experiential approach to teaching research skills. This may also reflect that the CEAR tool gives higher numeric values for experience vs. classroom learning.

Research and evidence-application skills are critical to the practice of medicine and therefore critical to medical education [2]. Despite the importance, there has been a lack of standardization in the way we teach and assess the many discrete skills required for both research and evidence-based practice. Further, the two concepts of research and evidence-application are easy to conflate with one another, which can lead to inadvertently teaching one instead of the other.

Previous systematic reviews of evidence-based practice education for healthcare professionals have highlighted the importance of using agreed-upon, validated, complete, and competency-focused tools for assessing outcomes of evidence-based practice education [18]. Similarly, there is a high degree of heterogeneity in the content and evaluation of curricula teaching research conduct in medical education [1].

Although tools and frameworks for assessing evidence-based practice education in medical education have been developed previously [6, 19], none of these explicitly address the overlap of evidence-application and research conduct skills. Similarly, while there is at least one existing tool to measure physician-scientist self-efficacy in a wide range of skills related to the conduct of clinical research [20], it does not assess skills that we consider to be foundational in the research process (i.e. Core domain skills), nor the overlap of these skills with evidence analysis. We propose the CEAR model as a valid theory to standardize the content and assessment of evidence-based practice and research curricula while acknowledging that evidence-based practice and research are distinct skill sets.

It would be valuable to conduct confirmatory factor analysis to confirm the validity of CEAR and demonstrate whether the skills in each Domain group together, and Domains separate from one another, as a follow-up to the exploratory factor analysis conducted in our registered dietitian sample [7]. However, we decided that medical students were not the right sample in which to conduct this analysis given that we expected (and observed) that students were mostly at the lower end of the skills. We would need a more diverse sample in terms of expected scores (i.e. some experts as well as novice students) to conduct confirmatory factor analysis.

Limitations of this analysis include the single site nature of the sample as well as the relatively small sample size. While we had robust participation at time 1 (313/340 possible participants), response rate decreased at later timepoints. While many students took the time 3 survey, few took all 3 surveys, limiting our ability to use repeated measures ANOVA. Our use of guaranteed gift card incentives at time 2 and 3 helped ensure that the sample did not reflect only those with an interest in research. Limitations of CEAR include its focus on human subjects research at the exclusion of basic science specific skills or examples. We are working on expanding CEAR to better capture this element of biomedical research in which many students participate as undergraduates. Further, the CEAR tool is a self-reported skills assessment and therefore cannot be relied upon as an objective assessment of research and evidence applications skills.

## Conclusions

Overall, we believe CEAR is a valid model for medical educators to use to conceptualize the separation of research and evidence application, the core skills required for both, and the sub-domains, in their development of curricula. Additionally, the CEAR tool may be useful for student assessment and curricular evaluation.

## Data Availability

The data that support the findings of this study are available from the corresponding author (RKH) upon reasonable request.
